# Shear Response of Glass Fibre Reinforced Polymer (GFRP) Built-Up Hollow and Lightweight Concrete Filled Beams: An Experimental and Numerical Study

**DOI:** 10.3390/polym12102270

**Published:** 2020-10-02

**Authors:** Sih Ying Kong, Leong Sing Wong, Suvash Chandra Paul, Md Jihad Miah

**Affiliations:** 1 School of Engineering, Monash University Malaysia, Bandar Sunway 47500, Malaysia; kong.sih.ying@monash.edu; 2 College of Graduate Studies, Universiti Tenaga Nasional, Kajang 43000, Malaysia; 3 Department of Civil Engineering, International University of Business Agriculture and Technology, Dhaka 1230, Bangladesh; suvashpl@iubat.edu; 4 Department of Civil Engineering, University of Asia Pacific, Dhaka 1205, Bangladesh; jihad.miah@uap-bd.edu

**Keywords:** GFRP composite, concrete infill, web stiffeners, web crippling, shear, Hashin damage

## Abstract

This paper investigated the static behaviour of glass fibre reinforced polymer (GFRP) built-up hollow and concrete filled built-up beams tested under four-point bending with a span-to-depth ratio of 1.67, therefore focusing their shear performance. Two parameters considered for hollow sections were longitudinal web stiffener and strengthening at the web–flange junction. The experimental results indicated that the GFRP hollow beams failed by web crushing at supports; therefore, the longitudinal web stiffener has an insignificant effect on improving the maximum load. Strengthening web–flange junctions using rectangular hollow sections increased the maximum load by 47%. Concrete infill could effectively prevent the web crushing, and it demonstrated the highest load increment of 162%. The concrete filled GFRP composite beam failed by diagonal tension in the lightweight concrete core. The finite element models adopting Hashin damage criteria yielded are in good agreement with the experimental results in terms of maximum load and failure mode. Based on the numerical study, the longitudinal web stiffener could prevent the web buckling of the slender GFRP beam and improved the maximum load by 136%. The maximum load may be further improved by increasing the thickness of the GFRP section and the size of rectangular hollow sections used for strengthening. It was found that the bond–slip at the concrete–GFRP interface affected the shear resistance of concrete–GFRP composite beam.

## 1. Introduction

Pultruded glass fibre reinforced polymer (GFRP) sections have shown potential application for bridges [1,2,3,4], building construction [5], and strengthening [6,7,8] due to the lightweight, high strength, and corrosion resistance characteristics. Pultruded GFRP is an orthotropic material with mostly unidirectional fibres aligned along the pultrusion direction. The Young’s modulus and compressive strength in the transverse direction are lower than those in the pultrusion direction due to this fibre architecture. This fibre architecture also causes the characteristic of low shear-to-compressive strength ratio of pultruded GFRP profiles. Therefore shear failure can occur prior to compressive failure when they are subjected to compression [9]. Therefore, pultruded GFRP sections are prone to web crippling failure, which includes web crushing, web buckling, and shear failure at the web–flange junctions when subjected to concentrated loads in the transverse direction.

Turvey and Zhang [10] found that the shear strength determined from web–flange junction specimens was significantly lower than the flat coupons extracted from this region due to the presence of a roving-rich core at the junction. Borowicz and Bank [11] reported shear failure at the upper web–flange junction of both pultruded GFRP I and wide flange sections subjected to three-point bending at the span-to-depth ratio of 4. The depth of beams considered was in the range of 152.4 mm to 304.8 mm. The maximum load increased by more than 35% when bearing plates were used to distribute the concentrated loads. A further study showed that the web buckling occurred at about 90% of the maximum load for the GFRP I beams with a depth of 609.5 mm tested under the same span-to-depth ratio before failed by web crushing [12]. Correia et al. [13] showed that one metre long pultruded GFRP (200 mm × 200 mm × 10 mm) I section failed by web crushing beneath the concentrated load when tested under three-point bending. Bai et al. [14] observed web–flange separation of three GFRP built-up sections tested under four-point bending and attributed the failure to high shear stress concentration in the web. Progressive damage was observed for four square hollow sections tested under end-two-flange (ETF) and interior-two-flange (ITF) loading conditions where failure initiated at the web–flange junction followed by buckling or crushing of the webs [15]. The results of 100.28 mm × 50.16 mm × 3.98 mm pultruded GFRP rectangular hollow sections tested under ETF and ITF with three different bearing lengths demonstrated that the maximum load fluctuated with the increased bearing length [16]. In contrast, the results of 99.5 mm × 49.72 mm × 5.8 mm pultruded GFRP I section indicated that the ultimate strengths typically reduced as the bearing length increased [17]. Fernandes et al. [18] investigated web crippling of four GFRP I sections with the depth ranged from 100 to 400 mm using two loading conditions, namely ETF and ITF under three different bearing lengths. The results showed that I sections were more prone to web buckling under ETF than ITF. The numerical study indicated that Hashin damage initiation criteria combined with the progressive damage model [19] could better predict the web crippling response compared to Tsai Hill model [20]. Finally, Wu et al. [21] showed that slenderness ratio, loading conditions, and specimens length could affect the failure mode. The pultruded GFRP C channels were more prone to web buckling with the increase of slenderness ratio and specimen length.

Web crippling could be effectively prevented by providing stiffeners at the web–flange junctions and infill for GFRP sections. Borowicz and Bank [22] showed that longitudinal angle stiffeners at the web–flange junctions delayed shear failure and increased the maximum load of GFRP I sections. Buckling of compressive flange and webs of the box beam could be prevented by placing stiffeners near the upper web–flange junctions [23]. Concrete infill prevented premature buckling and web crushing, thus increased the ultimate load by 100–141% compared to GFRP hollow beams tested under bending [24]. The ultimate load increased by 19% when the concrete compressive strength increased from 10 MPa to 43.5 MPa. Ferdous et al. [25] investigated the static response of concrete filled pultruded GFRP cellular and tubular sections used for modular retaining wall application. The results showed that concrete infill increased bending stiffness and maximum load by preventing local deformation such as crushing and buckling. The filled cellular sections failed by shear cracks at the upper web–flange junction.

There is limited research on shear behaviour of GFRP built-up hollow section and concrete filled beams. Some research has been conducted to study shear behaviour of concrete filled CFRP beams, hybrid concrete–GFRP beam and phenolic core-GFRP sandwich beams. Gautam and Matsumoto [26] studied the static response of hollow and concrete filled CFRP box beam subjected to transverse shear and axial loads. It was found that shear deformation contributed to 60% of total beam deflection and stiffness of concrete filled CFRP box beam was similar to hollow section due to slip between concrete–CFRP interface. A study on shear behaviour of hybrid concrete–GFRP box sections composite showed that all the specimens failed by longitudinal cracks at the top corners of the GFRP box section [27]. Ferdous et al. [28] studied the effects of beam orientation for phenolic core-GFRP sandwich beams at various shear span-to-depth ratios. Shear failure on GFRP skin was observed for beams tested with the span-to-depth ratio less than 2.2. However, the failure mode changed from the skin shear for a depth less than 40 mm to indentation when the depth increased to 80 mm.

This study investigated the shear behaviour of GFRP rectangular hollow and concrete filled built-up beams tested under four-point bending. Two parameters considered for hollow sections were longitudinal web stiffener and strengthening at the web–flange junction. Lightweight concrete was used as an infill to minimise weight gain. The experimental results indicated that hollow sections failed by web crushing at supports while concrete filled section failed by diagonal tension at the concrete core. Strengthening the web–flange junctions and concrete infill increased the maximum load of GFRP hollow sections. Finite element (FE) analyses adopting Hashin damage model achieved good agreement in terms of maximum load and failure mode compared to the experimental results. Finally, a parametric study was conducted to provide a better understanding of the effects of the thickness of GFRP sections and stiffness at the web–flange junction on the shear response of GFRP built-up beams. The parameters investigated were the size of C channel and square hollow sections (SHS), the thickness of the flanges, and the effectiveness of longitudinal stiffeners for GFRP beams with thinner webs.

## 2. Experimental Program

### 2.1. GFRP Beam Preparation

**Figure 1:**
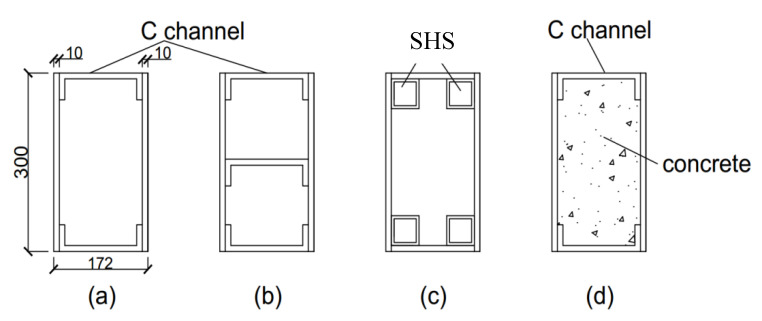
Glass fibre reinforced polymer (GFRP) built-up sections considered (**a**) rectangular hollow section (S1), (**b**) rectangular hollow section with longitudinal C channel stiffener at mid-height (S2), (**c**) rectangular hollow section reinforced using square hollow sections (SHS) at corners (S3), and (**d**) section filled with lightweight concrete (S4). All dimensions are in millimetres.

Four types of section prepared in this study are illustrated in Figure 1. Three parameters considered in this study were: (i) longitudinal web stiffeners, (ii) strengthening at the web–flange junction and (iii) concrete infill. The control beam was a rectangular hollow section with a dimension of 300 mm × 172 mm× 1600 mm which was fabricated using two pultruded GFRP C channels (152 mm× 42 mm× 9.5 mm), and two pultruded GFRP plates of 10 mm thickness bonded using DERAKANE 411-350 Epoxy Vinyl Ester Resin. The abbreviation of the control beam was S1 and its cross section was shown in Figure 1a. The total web thickness at the web–flange junction was 19.5 mm. The bonding surfaces were roughened using sandpapers, cleaned using acetone before the resin was applied. The epoxy resin was left hardened for 3 days. The second section was abbreviated as S2, it was fabricated with an additional C channel bonded to the webs at the centre along the longitudinal direction to reduce the buckling length of webs as shown in Figure 1b. The third section was abbreviated as S3, the stiffness at the web–flange junction was improved by replacing C channels with 50 mm × 50 mm × 5 mm square hollow sections (SHS) to join the flanges of 10 mm thickness to the 10 mm thick webs as shown in Figure 1c. The fourth section was abbrebirtaed as S4, it has the same configuration as the control beam with an additional lightweight concrete infill as shown in Figure 1d. Lightweight concrete was chosen to minimise weight gain due to the infill. The abbreviation of S1–4 will be used in the subsequent sections of this paper to refer to the respective type of beam.

### 2.2. Materials Properties and Experimental Setup

The mix proportion of lightweight concrete is presented in Table 1. Lightweight expanded clay aggregate (LECA) with a density of 400 kg/m^3^ was used to produce the lightweight concrete with a density of 1600 kg/m^3^. The slump of lightweight concrete was about 100 mm. During concrete casting, the GFRP hollow section was fixed vertically as concrete was filled into the void. Three concrete cylindrical specimens with a diameter of 100 mm and 150 mm height were prepared and cured for 28 days at the ambient temperature. The 28-day average compressive strength of lightweight concrete was 12 MPa.

The tensile strength of GFRP was determined based on ASTM standard [29]. The coupons were extracted from the undamaged webs in both longitudinal and transverse directions after beams testing. The tensile strength in the longitudinal direction was 257 MPa while it was 76 MPa in the transverse direction.

**Figure 2:**
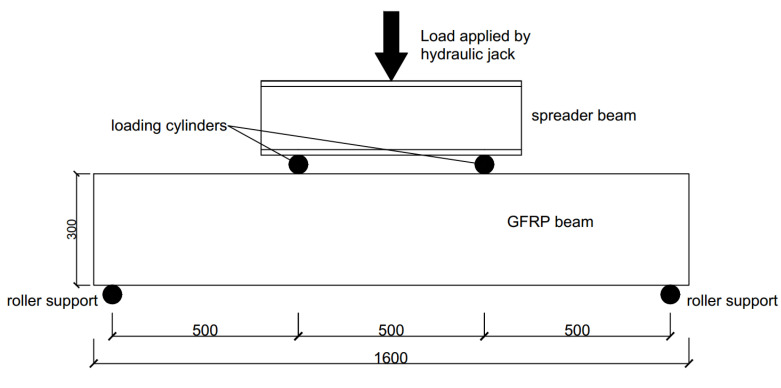
Four-point bending test for GFRP beams.

The GFRP beams were tested under four-point bending with the experimental setup illustrated in Figure 2. The chosen span-to-depth ratio was 1.67 in order to investigate the shear behaviours of GFRP hollow and filled composite beams. The load was applied by a Shimadzu hydraulic actuator with a capacity of 1000 kN at a displacement rate of 0.5 mm/min. The load and displacement were obtained from the record of the actuator. The displacement at mid-span was measured using a linear variable displacement transducer(LVDT).

## 3. Experimental Results

**Figure 3:**
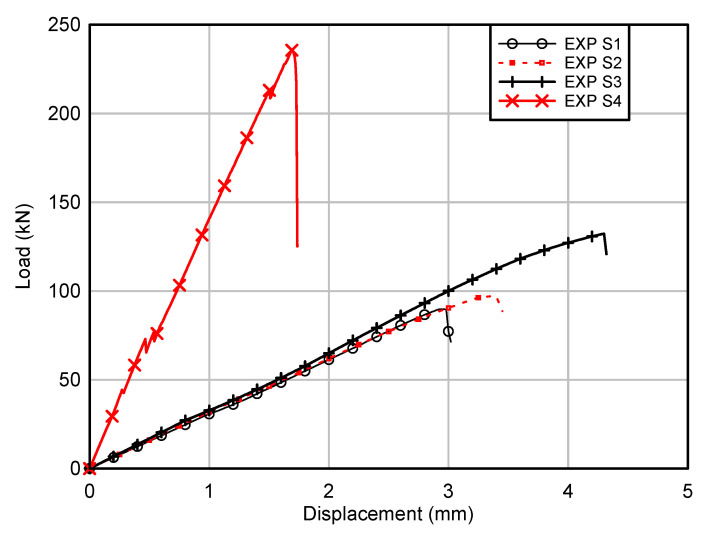
Experimental load–displacement curves of GFRP hollow and filled composite beams.

Load versus displacement response of all GFRP beams are depicted in Figure 3. It could be observed that the load increased linearly up to the failure load for both the control beam (S1) and hollow beam with longitudinal stiffener (S2). The GFRP beam with SHS at corners (S3) showed linear response initially up to 110 kN then the stiffness reduced gradually as the displacement further increased. For the concrete filled GFRP beam (S4), several minor drops of resistance could be observed before the failure load was reached, and the stiffness reduced slightly with each drop. All the hollow sections demonstrated similar stiffness, while the concrete infill significantly increased the stiffness of the GFRP beam, as demonstrated in Figure 3. Gautam and Matsumoto [26] found that there was no improvement of stiffness when comparing the experimental result of concrete filled CFRP box beam with a hollow CFRP beam tested under four-point bending. The authors attributed this phenomenon to the interface slip between concrete infill and CFRP box beam. However, Muttashar et al. [24] showed that stiffness increased by 25% when square GFRP hollow beams were filled with concrete. Furthermore, the study by Ferdous et al. [25] showed that concrete infill significantly improved stiffness of pultruded GFRP tubular sections and the bond–slip between concrete and GFRP was observed at the later loading stage. Thus, it could be deduced that concrete infill could improve the stiffness of pultruded GFRP hollow sections due to the composite action between GFRP sections and concrete infill.

**Figure 4:**
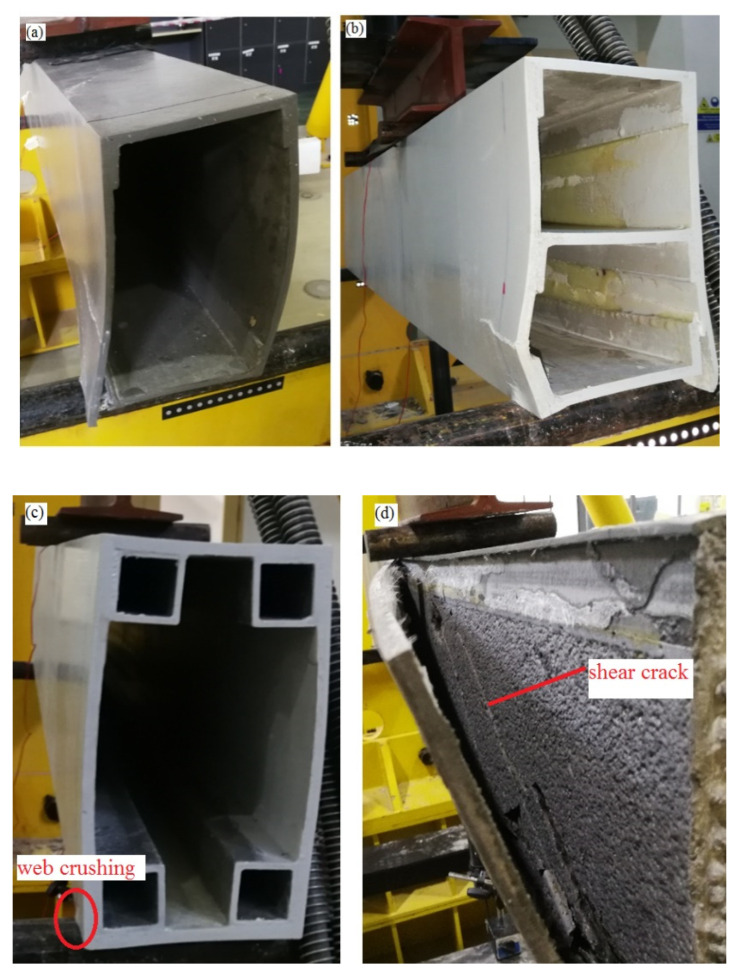
(**a**) web crushing followed by adhesive bonding failure at the supports of the control beam (S1), (**b**) web crushing followed by adhesive bonding failure at the supports of the GFRP beam with longitudinal stiffener (S2), (**c**) web crushing at the supports of the GFRP beam with SHS at corners (S3) and (**d**) diagonal tension failure of concrete core (S4).

The control beam (S1) showed the lowest maximum load of 90 kN, followed by the hollow beam with longitudinal stiffener (S2) of 97 kN. The maximum load of the beam with SHS at corners (S3) was 132 kN, while the concrete filled beam (S4) showed the highest maximum load of 236 kN. The difference of maximum load between the control beam (S1) and the hollow beam with longitudinal stiffener was small (S2), less than 8%, and this implied that the longitudinal stiffener was ineffective in improving the load capacity as the web buckling was not the governing failure mode for the control beam. Damage initiated by web crippling (web crushing at the supports) for both the S1 and S2 beams. This could be attributed to the low compressive strength in the transverse direction as the glass fibre mainly aligned in the longitudinal direction due to the pultrusion process. As the displacement of actuator further increased, the adhesive bonding between webs and the bottom C channel failed as depicted in Figure 4a,b, causing a brittle failure in these beams. Wu and Bai [15] observed the progressive failure process for pultruded GFRP square sections, as the load could be resisted by webs after initial failure at the web–flange junction.

The maximum load of the beam with SHS at corners (S3) was 47% higher than the control beam (S1). The SHS improved the bearing capacity of the flanges as the thickness of the flanges was increased by 5 mm at the web–flange junction compared to the control beam. The use of SHS could also improve the rotational stiffness at the web–flange junction due to the higher rotational rigidity of SHS compared to the C channel. The study by Borowicz and Bank [22] showed that the maximum load increased by 50–70% when GFRP angles were used to strengthen the web–flange junction of I sections. The failure mode of this beam was similar to the control beam (S1), where web crushing initiated at the supports followed by an adhesive failure between webs and SHS at the supports.

The lightweight concrete infill improved the maximum load by 162%. Concrete infilled beam showed the highest load improvement among all the options considered. However, the lightweight concrete infill increased the weight of GFRP beam significantly. The concrete infill eliminated the web crushing of GFRP section as the concentrated force at the supports could be distributed to the concrete infill. This beam demonstrated brittle behaviour at the maximum load, and the damage is shown in Figure 4d. It was believed that the diagonal tension failure (shear crack) in the plain concrete core triggered the failure of the beam and then followed by the failure of adhesive bonding between webs and C channels. As the span-to-depth ratio for this beam was 1.67, the inclined crack observed in the plain lightweight concrete core between the supports and loading points was similar to the failure mode observed for the concrete beams without stirrups tested under span-to-depth ratio in the range of 1 to 2 [30].

## 4. FE Modelling

The commercial finite element software, Abaqus, was used in this study. The GFRP beams were modelled using shell elements where different shell thicknesses were defined at the web–flange junctions to correctly model variation of web thickness due to joining C channels or SHS to the webs. The GFRP sections were discretised using four-node reduced integration shell elements (S4R) with a mesh size of 10 mm. In contrast, the concrete infill was discretised using eight-node reduced integration continuum 3D solid elements (C3D8R) with a mesh size of 20 mm. The loading cylinders and supports were modelled using solid elements (C3D8R) and assigned with elastic material properties. The surface to surface contact was used to model the interaction between the cylinders and the GFRP beams. For interaction between concrete infill and GFRP section, it was found that bond–slip and normal separation between GFRP webs and concrete infill should be considered in order to predict shear failure in the concrete infill accurately. Therefore, the surface to surface contact was used. A similar observation was reported for CFRP box beams filled with concrete [26], and concrete filled pultruded GFRP sections [25]. The top and bottom flanges were assumed to have perfect bonding with the lightweight concrete core by using tie constraint. The loading was applied by assigning a constant displacement to the loading cylinders. To model the roller supports, vertical displacement is restrained over 10 mm region of the GFRP bottom flange to avoid unrealistic stress concentration.

### 4.1. Hashin Damage Model

Hashin damage initiation criteria could be used to predict anisotropic damage in elastic-brittle materials such as GFRP by considering four different failure modes, namely (i) fibre tension ($F_{f}^{t}$), (ii) fibre compression ($F_{f}^{c}$), (iii) matrix tension ($F_{m}^{t}$), and (iv) matrix compression ($F_{m}^{c}$). A previous numerical investigation showed that the Hashin damage model could accurately predict the web crippling failure of GFRP I sections, and it provided more reliable results than the Tsai-Hill criterion [20]. The general form of damage initiation criteria are:

(1) $$F_{f}^{t}={\left(\frac{\sigma_{11}}{S_{t,1}}\right)}^{2}+\alpha {\left(\frac{\sigma_{12}}{S_{12}}\right)}^{2}\mathrm{for}\;\mathrm{fibre}\;\mathrm{in}\;\mathrm{tension},\;\sigma_{11}\geq 0$$

(2) $$F_{f}^{c}={\left(\frac{\sigma_{11}}{S_{c,1}}\right)}^{2}\;\mathrm{for}\;\mathrm{fibre}\;\mathrm{in}\;\mathrm{compression},\;\sigma_{11}<0$$

(3) $$F_{m}^{t}={\left(\frac{\sigma_{22}}{S_{t,2}}\right)}^{2}+{\left(\frac{\sigma_{12}}{S_{12}}\right)}^{2}\;\mathrm{for}\;\mathrm{matrix}\;\mathrm{in}\;\mathrm{tension},\;\sigma_{22}\geq 0$$

(4) $$F_{m}^{c}={\left(\frac{\sigma_{22}}{2S_{23}}\right)}^{2}+\left[{\left(\frac{S_{c,2}}{2S_{23}}\right)}^{2}-1\right]\frac{\sigma_{22}}{S_{c,2}}+{\left(\frac{\sigma_{12}}{S_{12}}\right)}^{2}\;\mathrm{for}\;\mathrm{matrix}\;\mathrm{in}\;\mathrm{compression},\;\sigma_{22}<0$$

where $\sigma_{11},\sigma_{22}$ σ_22_, and σ_12_ are the effective stress tensors in the longitudinal, transverse, and in-plane shear. S_t,1_ and S_c,1_ denote the tensile and compressive strength in the longitudinal direction, S_t,2_ and S_c,2_ denote tensile and compressive strength in the transverse direction, while S_12_ and S_23_ denote shear strength in the longitudinal and transverse direction. α is a coefficient (either 0.0 or 1.0) to take into account the contribution of in-plane shear stress on the damage of fibre in tension. The default value of 0.0 was adopted in this study as the fibre tension failure was not critical for web crippling failure. Transverse compressive and in-plane shear strength governs the ultimate load of the GFRP section failed by web crippling [20]. The longitudinal and transverse tensile strength was defined based on the experimental results of 257 MPa and 76 MPa, respectively. The longitudinal and transverse compressive strength was obtained by calibrating the FE model using the experimental results of the control beam. The longitudinal compressive strength was defined as 137 MPa, and the transverse compressive strength was 102 MPa. The elastic modulus in the longitudinal and transverse directions was 12.5 GPa and 4.5 GPa, respectively.

Once any of the damage criteria specified above is met, the GFRP degrades based on damage evolution defined in the finite element analyses. In this study, the energy based damage evolution was chosen by defining fracture energy for each failure mode. The ultimate load of GFRP sections failed by web crushing is very sensitive to the fracture energy associated with matrix compression, and this value could be calibrated to improve the accuracy of FE models [20]. By defining a higher value of fracture energy, the maximum load could be increased as the damage is distributed to a larger area before the GFRP section failed. In the absence of experimental results, this value should be carefully calibrated so that the FE predicted failure mode matches the experimental observation to avoid overestimation of the ultimate load. The fracture energy of 7 N/mm was chosen for matrix compression based on the calibration of the FE model used in this study. Damage stabilisation was included by defining viscosity coefficients for each damage criterion to improve the convergence of the numerical analysis. It is worth noting that viscosity coefficients could affect the ultimate load, and the value of 1 × 10^−5^ was adopted in this study [20].

### 4.2. Concrete Damaged Plasticity Model

Concrete damaged plasticity (CDP) model is a continuum, plasticity based damage model suitable for both plain concrete and reinforced concrete structures. It could be used to model concrete structures subjected to monotonic, cyclic and dynamic loading under low confining pressures. It considers isotropic damaged elasticity in combination with nonassociated multi-hardening plasticity in tension and compression to model irreversible damage of concrete during cracking and crushing. It assumes nonassociated potential plastic flow based on the Drucker-Prager hyperbolic function, which requires a definition of parameters such as dilation angle (ψ) and flow potential eccentricity (ε). For the dilation angle, a typical value of 31° was adopted in this study. From literature, a wide range of dilation angle, from 5° [31] to 40° [32] has been adopted to simulate shear behaviour of reinforced concrete structures as it was found that reducing dilation angle resulted in a lower ultimate load. A default flow potential eccentricity (ε) of 0.1 is recommended by AbaqusTheory guide [33], which implies the same dilation angle is used over a wide range of confining pressure. The yield function considers different evolution of strength controlled by hardening variables in tension and compression. Two ratios considered in the yield function are (i) ratio of initial equibiaxial compressive yield stress to initial uniaxial compressive yield stress (f_bo_/f_co_) and (ii) ratio of the second stress invariant on the tensile meridian to the compressive meridian (K_c_). The default values for these parameters are shown in Table 2. This material model includes a viscosity parameter to improve the convergence rate in the softening region. However, it was found that it led to unrealistic ductility of plain concrete core used in this study, where the shear crack distributed over a large region of concrete instead of discrete shear crack observed at the maximum load. Therefore, the viscosity parameter of 0.0 was adopted.

The tensile strength ($f_{\mathrm{lctm}}$), Young’s modulus ($E_{\mathrm{lci}}$), and stress–strain ($\sigma_{c},{\;\varepsilon }_{c}$) relationship of the lightweight concrete were determined based on Model code 2010 [34]:

(5) $$f_{\mathrm{lctm}}=\left(0.4+\frac{0.6\rho }{2200}\right)\times 0.3{\left(f_{\mathrm{ck}}\right)}^{2/3}$$

(6) $$E_{\mathrm{lci}}={\left(\frac{\rho }{2200}\right)}^{2}\times 21.5\times 10^{3}\times {\left(\frac{f_{\mathrm{cm}}}{10}\right)}^{1/3}$$

(7) $$\frac{\sigma_{c}}{f_{\mathrm{lcm}}}=-\left(\frac{k\eta -\eta^{2}}{1+\left(k-2\right)\eta }\right)\;\mathrm{for}\;\left|\varepsilon_{c}\right|<\left|\varepsilon_{c,\lim}\right|$$

where ρ is the oven dry density, f_ck_ and f_cm_ are concrete characteristic strength and mean strength, respectively, k is the plasticity number, and η is $\varepsilon_{c}/\varepsilon_{c1}$.

Tension stiffening was modelled using linear stress–crack opening relationship based on the fracture energy (GF) of lightweight concrete. For plain concrete or concrete with a low percentage of reinforcement, the specification of stress–strain relation for tension stiffening leads to mesh sensitivity where the analysis results vary as the mesh is refined due to localisation of discrete cracks [33]. The stress–crack opening approach was adopted to overcome the mesh dependency problem [35].

## 5. Validation and Parametric Study

### 5.1. Validation of FE Models

**Figure 5:**
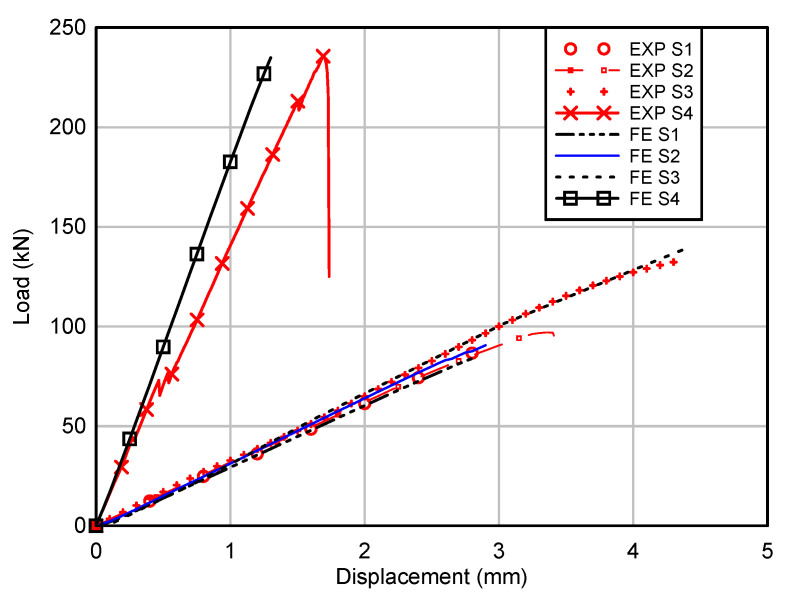
Comparison between experimental and FE predicted load–displacement response of GFRP hollow and filled composite beams.

**Figure 6:**
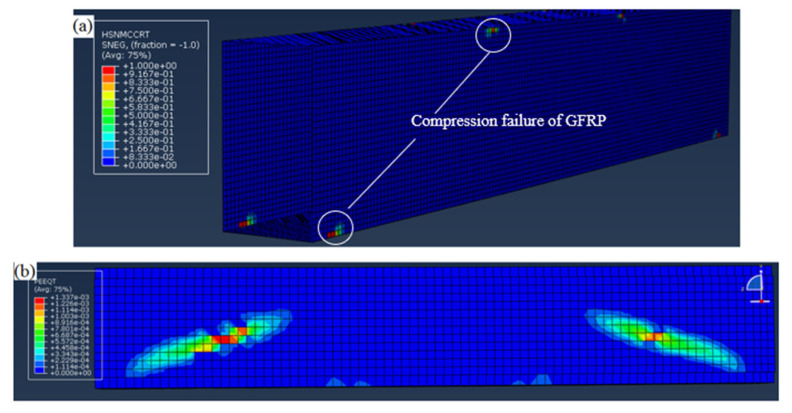
Damage predicted by FE models (**a**) crushing of GFRP at the web–flange junction for the control beam (S1) and (**b**) diagonal tension failure of the plain lightweight concrete core.

The simulation results are compared to the experimental load–displacement relationships in Figure 5. The predicted stiffness of hollow sections corresponded very well with the experimental results. The predicted maximum load for all the beams was similar to the experimental results. For the control beam (S1), the predicted maximum load was 84 kN, only 6.7% lower than the experimental result. The maximum load predicted for the hollow beam with longitudinal stiffener (S2) was 91 kN, only 6.2% lower than the maximum experimental load. The predicted load for S1 and S2 was similar, indicating that the internal C stiffener only has minor effects on the load capacity as the beam failed by web crushing. The maximum load predicted for the beam with SHS at corners (S3) was 140 kN, and it was 6.1% higher than the experimental results. All FE models of hollow sections predicted similar damage with the experimental observations, where the damage initiated at the web–flange junction at the supports and the loading points as exemplified by the damage of the control beam (S1) shown in Figure 6a. The “HSNMCCRT” in the legend of Figure 6a refers to the matrix compressive initiation criterion.

For the concrete filled GFRP beam (S4), the FE model using tie constraint for interaction between concrete core and GFRP section predicted a maximum load of 460 kN which was significantly higher than the experimental result of 236 kN. This implied that the interaction at concrete–GFRP interface governed the failure load of the composite section. In this study, the interaction between GFRP webs and concrete was modelled using the surface to surface contact, a simplified modelling technique which ignored the initial concrete bonding. This contact algorithm allows normal separation of contact surfaces and shear transfer at the interface. However, the definition of the surface to surface contact resulted in the underestimation of the maximum load as the shear stress was mainly resisted by the concrete core causing concrete failed prematurely. It should be noted that GFRP webs acted as the reinforcement of concrete before bonding failure. In order to take into account the effects of reinforcement contributed by GFRP, the tensile strength of lightweight concrete was increased from 0.6 MPa (determined using Model code 2010 [34]) to 1.5 MPa. The FE predicted maximum load was 236 kN which agreed very well with the experimental results of 236 kN. Furthermore, the predicted failure mode matched the experimentally observed shear crack, as shown in Figure 6b. The “PEEQT” in the legend of Figure 6b refers to equivalent plastic strain in uniaxial tension. It is worth noting that this simplified technique could be used only if the experimental results of concrete filled GFRP beams are available. The bond–slip response of concrete should be included in the FE model using cohesive elements in Abaqus whenever the experimental bond strength vs slip is available.

### 5.2. Parametric Study

Using the validated FE model, three parameters further investigated were (i) thickness of web and flange, (ii) effectiveness of longitudinal stiffener on slender GFRP built-up section, and (iii) size of SHS at corners.

#### 5.2.1. Effects of GFRP C Channel and Web Thickness

**Figure 7:**
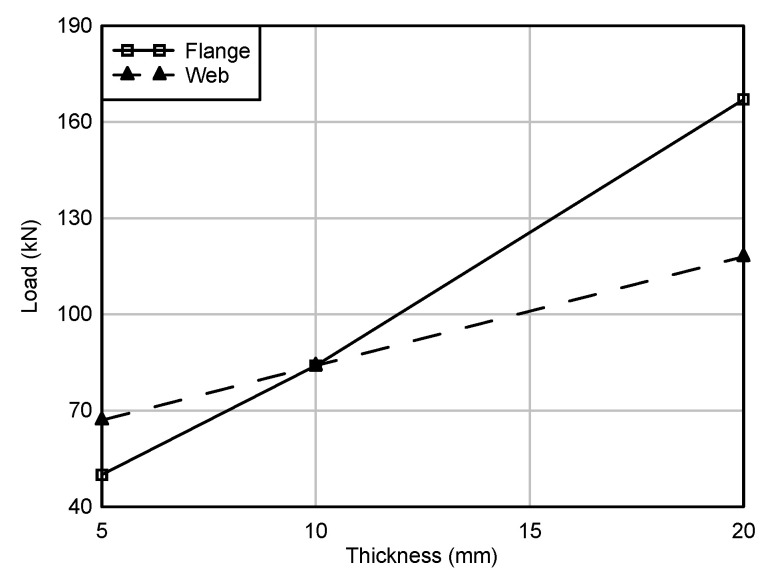
Maximum load variation due to varying web and C channel thickness.

Three thicknesses considered for web and C channel were 5 mm, 10 mm, and 20 mm. The predicted maximum load by varying the thickness of these components is shown in Figure 7. It could be observed that varying the thickness of the C channel showed a more significant effect on the maximum load compared to the web thickness. For instance, increasing the C channel thickness from 10 mm to 20 mm resulted in a load increment of 98% while the maximum load increased by only 40% when the web thickness increased from 10 mm to 20 mm. As the thickness of the C channel increased, the rotation stiffness at the web–flange junction increased, and the concentrated load at the supports could be distributed to a larger area of webs through the thicker flange. Both phenomena contributed to maximum load improvement. While increasing the web thickness only increased the bearing strength of the webs, but the rotational stiffness still controlled by the thickness of the C channel. The failure mode observed was web crushing for the all thicknesses considered in this study.

#### 5.2.2. Effectiveness of Longitudinal Stiffener to Restrain Web Buckling

**Figure 8:**
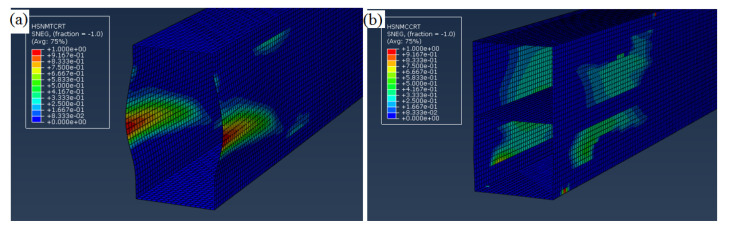
Failure mode of GFRP built-up beam with 2.5 mm web thickness (**a**) hollow section and (**b**) beam with longitudinal stiffener.

The effectiveness of GFRP longitudinal stiffener in restraining the web buckling of GFRP built-up closed section was investigated by reducing the web thickness to 2.5 mm. The web buckling could be observed for the hollow section, as shown in Figure 8a, and the predicted maximum load was 15 kN. The hollow GFRP beam failed by a longitudinal crack near mid-height of each web started from the end of the beam due to the web buckling. The longitudinal stiffener could effectively restrain the web buckling and improved the maximum load to 47 kN (213%). The stiffened beam failed by web crushing at the supports and loading points, as shown in Figure 8b. From these observations, it could be concluded that the GFRP C channel could be used as the longitudinal web stiffener to prevent buckling of slender GFRP webs.

#### 5.2.3. Effects of Different SHS Sections at Corners

**Figure 9:**
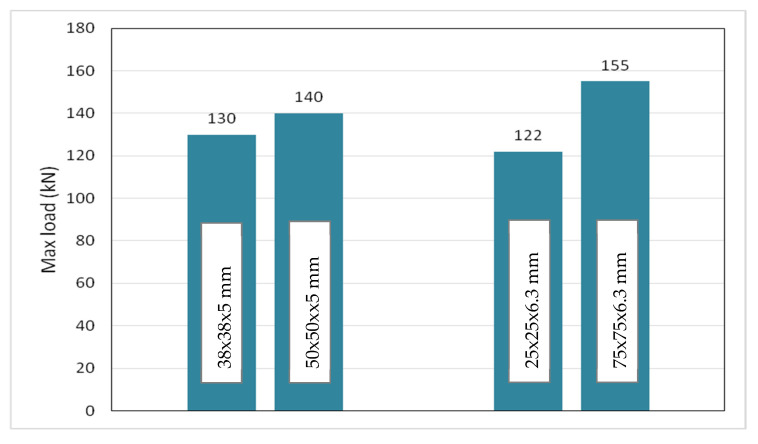
Maximum load of GFRP hollow built-up section using different sizes of square hollow section.

In this section, two geometry parameters investigated were the thickness and height of SHS. Two thicknesses studied were 5 mm and 6.3 mm. Two sizes of SHS considered for 5 mm thickness were 38 mm × 38 mm × 5 mm and 50 mm × 50 mm × 5 mm, while 25 mm × 25 mm × 6.3 mm and 75 mm × 75 mm × 6.3 mm were studied for 6.3 mm thickness. The maximum load for the GFRP built-up section using different SHS is illustrated in Figure 9. It could be observed that the maximum load increased as the height of SHS increased, which could be attributed to the higher rotational stiffness at the web–flange junction as the height of SHS increased. The GFRP beam with 25 mm × 25 mm × 6.3 mm SHS at corners showed a slightly lower maximum load than the beam using 38 mm × 38 mm × 5 mm SHS even though it has a greater thickness at the web–flange junction. These results demonstrated that the height of SHS has a more significant influence on the maximum load than the thickness of SHS.

## 6. Conclusions

In this study, the shear behaviour of GFRP hollow and concrete filled built-up beams was studied experimentally and numerically. The rectangular hollow built-up section was fabricated using two pultruded GFRP C channels, and two pultruded GFRP plates bonded using epoxy adhesive. The parameters considered including longitudinal web stiffener, strengthening at the web flange junction and lightweight concrete infill. Based on the experimental and simulation results, the following conclusions were drawn:

(a) The GFRP hollow built-up sections failed by web crushing at the supports due to orthotropic material properties of GFRP. The rotational stiffness at the web–flange junction governed the maximum load of the built-up beam, and increasing the thickness of the C channel could improve the maximum load more effectively than increasing the web thickness. The longitudinal C channel stiffener could prevent the web buckling of slender GFRP hollow built-up section, and the maximum load increased by 136% for the web thickness considered in the numerical study.
(b) The built-up section with square hollow sections at corners demonstrated 47% load improvement due to the enhancement of stiffness at the web–flange junction. Increasing the height and thickness of SHS could improve the maximum load of the built-up section. The height of the SHS section showed a greater influence on the maximum load than the thickness of SHS.
(c) Concrete infill improved the stiffness and maximum load by preventing web crushing failure of GFRP section. It achieved the highest maximum load increment of 162% among the parameters considered in this study. It was found that the maximum load was influenced by the bond–slip behaviour at the concrete–GFRP interface, and the failure was caused by the shear crack in the plain concrete core.

Even though concrete infill is the most effective alternative for improving the stiffness and maximum load, the numerical simulation for shear behaviour of concrete filled GFRP composite beam is very challenging due to the bond–slip at the concrete–GFRP interface. More research is needed to study the interaction at concrete–GFRP interface subjected to shear and normal force, as it is the key information required to improve the capability of the FE model.

## Figures and Tables

**Table 1:** Lightweight concrete mix proportion.

	Proportion
Cement (kg/m³)	424
Sand (kg/m³)	847
LECA (kg/m³)	327
Water (kg/m³)	170

**Table 2:** Concrete damage plasticity parameters.

Dilation Angle, ψ	Eccentricity, ε	f_bo_/f_co_	K_c_	Viscosity Parameter
31	0.1	1.16	0.667	0.0
